# The prevalence of functional urinary incontinence and its association with comprehensive geriatric assessment parameters in older women

**DOI:** 10.1007/s40520-025-03228-9

**Published:** 2025-11-14

**Authors:** Elif Meseci, Pinar Soysal, Irem Tanriverdi, Satı Betul Beydilli, Ozge Pasin, André Hajek, Masoud Rahmati, Lee Smith

**Affiliations:** 1 Clinic of Obstetrics and Gynecology, Acıbadem Kozyatağı Hospital, İstanbul, Türkiye; 2 Department of Geriatric Medicine, Faculty of Medicine, Bezmialem Vakif University, Istanbul, Türkiye; 3 Department of Internal Medicine, Faculty of Medicine, Bezmialem Vakif University, Istanbul, Türkiye; 4 Department of Biostatistics, Hamidiye Medical Faculty, Health Sciences University, Istanbul, Türkiye; 5 Department of Health Economics and Health Services Research, University Medical Center Hamburg-Eppendorf, Hamburg Center for Health Economics, Hamburg, Germany; 6 CEReSS-Health Service Research and Quality of Life Center, Assistance Publique-Hopitaux de Marseille, Aix-Marseille University, Marseille, France; 7 Department of Physical Education and Sport Sciences, Faculty of Literature and Human Sciences, Lorestan University, Khoramabad, Iran; 8 Department of Physical Education and Sport Sciences, Faculty of Literature and Humanities, Vali-E-Asr University of Rafsanjan, Rafsanjan, Iran; 9 Centre for Health Performance and Wellbeing, Anglia Ruskin University, Cambridge, UK; 10 Department of Public Health, Faculty of Medicine, Biruni University, Istanbul, Türkiye

**Keywords:** Comprehensive geriatric assessment, Urinary incontinence, Older adults

## Abstract

**Aim:**

The study aims to examine the prevalence of functional urinary incontinence (FUI) and its comparative frequency with other types of incontinence. Moreover, this study also aims to explore its relationship with key geriatric assessment parameters in older women.

**Methods:**

Older women over the age of 65 years from one geriatrics outpatient clinic were included in the cross-sectional study. UI subtypes were classified based on participants’ responses to the International Consultation on Incontinence Questionnaire–Urinary Incontinence Short Form. FUI is the involuntary leakage of urine resulting from physical or cognitive limitations in reaching or using toilet facilities. The relationships between UI subtypes and comprehensive geriatric assessment parameters were determined

**Results:**

The study included 1628 participants (mean age 79.6 ± 8.2 years). Prevalence rates were identified as follows: control group (no continence) (37.2%), Urge UI (31.9%), Stres UI (4.6%), FUI (7.6%), Mixt UI (urge and stress, 12.3%), Urge UI and FUI (4.9%), and Stress UI with FUI (1.5%). In multınominal lojistik regression, Basic Activities of Daily Living scores were low across all types of urinary UI (p<0.05). Patients with FUI exhibited significantly lower Mini Nutritional Assessment scores, Tinetti balance and gait scores, and handgrip strength compared to those with other UI subtypes, whereas their Timed Up and Go times and age were higher (p<0.05).

**Conclusion:**

FUI is associated with several geriatric conditions, including increased functional dependency, nutritional deterioration, reduced muscle strength, and impaired balance and gait functions. Therefore, when approaching an older woman with incontinence, it is essential to be aware of FUI, rather than focusing solely on Urge UI or Stress UI.

**Supplementary Information:**

The online version contains supplementary material available at 10.1007/s40520-025-03228-9.

## Introduction

Geriatric syndromes are multifactorial health conditions that occur when the accumulated effects of impairments in multiple systems render an older person vulnerable to situational challenges [1]. Urinary incontinence (UI) is one of the most prevalent clinical conditions among the geriatric syndromes and represents a significant health concern in older adults due to its wide-ranging physical and psychosocial consequences [2, 3]. UI is a common condition among older women, and is defined by the International Continence Society (ICS) as a lower urinary tract symptom characterized by the complaint of involuntary leakage of urine from the urethral opening [4]. The prevalence of urinary incontinence in this population has been reported to vary considerably across studies, ranging from 14.4% to 80% [5, 6]. The three primary subtypes are stress urinary incontinence (SUI), urge urinary incontinence (UUI), and mixed urinary incontinence (MUI), each with distinct pathophysiological mechanisms [7]. SUI is defined as the involuntary leakage of urine during physical exertion or activity such as coughing, sneezing, or exercise. UUI refers to involuntary urine loss associated with a sudden and compelling urge to void that is difficult to defer. MUI is characterized by a combination of both stress- and urgency-related symptoms [8]. In addition to these common forms, overflow incontinence is another clinically relevant type, characterized by involuntary leakage due to bladder overdistention, often resulting from detrusor underactivity or bladder outlet obstruction [9]. Another important, yet often underrecognized, subtype is functional urinary incontinence (FUI), which occurs despite normal lower urinary tract function. This form is typically caused by physical or cognitive impairments that delay or prevent timely access to toileting. Conditions such as osteoarthritis, sarcopenia, and Parkinson’s disease may limit mobility or compromise an individual’s ability to recognize or appropriately respond to bladder signals, thereby increasing the risk of functional incontinence [10, 11].

UI is associated with a poor quality of life and mobility problems, presence of cognitive problems, dependency and female sex [12, 13]. UI in older women is not merely a physiological condition characterized by skin irritation, falls, or urinary tract infections; it also exerts profound psychological consequences such as depression, anxiety, and social withdrawal. Beyond the individual level, it poses significant sociological challenges—often precipitating the need for institutional care—and imposes a substantial economic and systemic burden on healthcare services. As such, UI should be regarded as a complex geriatric syndrome with far-reaching implications that transcend the boundaries of physical health [14]. Although numerous studies have focused on stress, mixed, and urge urinary incontinence in women, research specifically addressing incontinence subtypes in the geriatric population remains limited. In particular, FUI, which increases in prevalence with advancing age, has often been overlooked, resulting in a significant gap in our understanding of its characteristics and clinical implications in older women.

Geriatric syndromes refer to clinical conditions that do not fit neatly into traditional disease classifications but are highly prevalent in older adults, often arising from the interaction of multiple physiological systems [1]. These conditions commonly threaten an individual’s independence and functional capacity. UI is closely associated with geriatric syndromes due to its multifactorial nature and its interplay with both cognitive and physical impairments, as well as environmental influences [15]. Cognitive decline, dementia, delirium, sarcopenia, mobility limitations, and polypharmacy are among the common components of geriatric syndromes that can contribute to the onset and worsening of UI [1]. Therefore, the evaluation and management of UI in older women should not be limited to urogynocologic considerations alone but should be approached within the framework of a comprehensive geriatric assessment.

Given this background this study aims to raise awareness about FUI in the geriatric population by examining the prevalence of FUI and its comparative frequency with other types of incontinence. Moreover, this study also aims to explore its relationship with key geriatric assessment parameters in older women.

## Methods

The research methods and all procedures conducted involving human participants adhered to the ethical guidelines set forth by the institutional and/or national research committee (Ethics Committee of Bezmialem Vakif University—E-35700536-108.99.99-170908/30.10.2024) and were in compliance with the 1964 Helsinki Declaration, including its subsequent amendments or comparable ethical standards.

A total of 1.628 older females over the age of 65 years from one geriatrics outpatient clinic in Istanbul, Turkiye were included in the cross-sectional study according to the inclusion and exclusion criteria. Patients were excluded from the study if they were male, presented with acute incontinence symptoms (such as acute cystitis or newly initiated medication side effects), had moderate to severe dementia, suffered from life-threatening conditions (such as cancer or advanced heart failure, acute strokes, blood infections), refused to undergo a comprehensive geriatric assessment (CGA), experienced communication difficulties (e.g., due to hearing impairment), or had incomplete medical records. Those whose clinical condition (such as delirium) prevented them from undergoing CGA were also not included.

### Comprehensive geriatric assessment (CGA)

CGA was performed, and data regarding the participants were gathered through interviews carried out by a multidisciplinary team, including a geriatrician, internal medicine doctor, and gerontologist. These professionals conducted interviews with the family members or caregivers of each patient enrolled in the study.

The study collected demographic and clinical data, including participants’ age, gender, and body mass index (BMI), the latter calculated using the formula weight in kilograms divided by height in square meters. It also recorded the presence of various comorbidities such as dementia, hypertension, diabetes mellitus, coronary artery disease, congestive heart failure, chronic obstructive pulmonary disease (COPD), Parkinson’s disease, cerebrovascular event, and osteoarthritis. Medication use, along with the total number of medications, was documented.

For the nocturia variable, the question, “Generally, during the past 30 days, how many times do you usually urinate after you have gone to sleep at night until the time you got up in the morning?’’ was used. The wording of the question shows strong agreement with the ICS definition of nocturia [16]. Calf circumference was measured with a millimeter-marked tape measure while participants were lying supine with the left knee elevated and the calf at a right angle to the thigh. Upper arm circumference was measured at the midpoint between the acromion and olecranon processes using a flexible measuring tape. The participant’s arm waws relaxed by their side, and the tape was applied snugly around the arm, with the measurement recorded to the nearest millimeter [17].

Mini-Mental State Examination, and Geriatric Depression Scale-15 was applied for neurocognitive and mood evaluation, Basic and Instrumental Activities of Daily Living (BADL and IADL) for functional evaluation, Tinetti Performance-Oriented Assessment of Mobility, and Timed Up and Go Test (TUG) for mobility evaluation, Mini-Nutritional Assessment (MNA) scale for nutritional evaluation, The Council on Nutrition Appetite Questionnaire for loss of appetite, and EAT-10 for dysphagia. Insomnia and daytime sleepiness were assessed using the Insomnia Severity Index (ISI) and Epworth Sleepiness Scale, respectively. SARC-F was used for screening of sarcopenia. Handgrip strength was assessed using a Jamar dynamometer, with the average of three measurements recorded. All the appropriate references of CGA were stated in our previous articles [18–20].

## Assessment of urinary incontinence and its subtypes

UI subtypes were classified based on participants’ responses to the International Consultation on Incontinence Questionnaire – Urinary Incontinence Short Form (ICIQ-UI SF) [21]. A diagnosis of SUI was assigned to individuals who reported urine leakage during activities such as coughing, sneezing, or physical exertion/exercise, provided that they did not endorse the item indicating leakage prior to reaching the toilet. Conversely, UUI was defined by the presence of urine leakage before reaching the toilet, in the absence of responses suggestive of stress-related incontinence. Participants who endorsed both stress-related and urgency-related items were categorized as having MUI. In cases where participants responded negatively to both SUI- and UUI-related items but reported challenges with controlled or comfortable mobility, further evaluation was conducted to assess for FUI. For this purpose, the DIPPERS assessment was employed, a structured approach designed to identify potentially reversible contributors to incontinence. This assessment comprised the following components: Delirium, Infection, Pharmaceuticals, Psychological disorders, Excessive fluid intake, Restricted mobility, and Stool impaction—collectively referred to as the DIPPERS framework [22].

## Laboratory findings

The laboratory assessments conducted to evaluate the biochemical, metabolic, and nutritional status of the patients encompassed a range of tests, including the complete blood count, kidney and liver function markers, cholesterol levels, thyroid stimulating hormone, HbA1c, as well as measurements of vitamin B12, folic acid, and vitamin D. The Glomerular Filtration Rate (GFR) was determined using the Modification of Diet in Renal Disease (MDRD) formula. All biochemical analyses were carried out using the Diagnostic Modular Systems autoanalyzer (Roche E170 and P-800).

### Statistical analysis

Descriptive statistics for categorical variables in the study were presented as numbers and percentages, while descriptive statistics for continuous variables were given as mean, standard deviation, median, minimum, and maximum. Pearson’s chi-square and Fisher-Freeman-Halton tests were used to evaluate the relationships between categorical variables. The normality of continuous variables was assessed using the Kolmogorov-Smirnov test. The Kruskal-Wallis test was used for comparing means across more than two independent groups. For multiple comparisons, the Dunn test was employed as a post hoc analysis method. Variables found to be significant in univariate analyses were included simultaneously in the model using multinomial logistic regression analysis for multivariate analysis, and their effects were evaluated. Odds ratios and 95% confidence intervals were calculated to observe the effect of each variable. The level of statistical significance was set at 0.05, and all calculations were performed using the Statistical Package for the Social Sciences (SPSS) (Version 28.0, Armonk, NY, IBM Corp.).

## Results

The study included 1628 participants (mean age 79.6 ± 8.2 years). Prevalence rates were identified as follows: control group (no continence) (37.2%), UUI (31.9%), SUI (4.6%), FUI (7.6%), MUI (urge and stress, 12.3%), UUI and FUI (4.9%), and SUI with FUI (1.5%). When the clinical and functional characteristics of the subgroups were compared, statistically significant differences were found in terms of basic parameters such as age, physical performance level, cognitive capacity and comorbidity burden (overall *p* < 0.001 for all comparisons). Post-hoc pairwise comparisons following multiple group analyses for each variable revealed the existence of heterogeneous clinical profiles among incontinence subtypes. The comparative results showing the significant differences between the groups based on relevant clinical criteria, the direction of these differences and their statistical significance levels are summarized in Table 1.

**Table 1:** General characteristic features according to the types of incontinence

Variable	Control Group	Urge UI (UUI)	Stress UI (SUI)	Functional UI (FUI)	Mixt UI	SUI & FUI	UUI & FUI	*p*-value
**Age (years)**	79.6 ± 8.1 80 (65–100)	83.9 ± 81.3 81 (68–96)	78.3 ± 7.8 79 (69–93)	83.80 ± 7.02 84 (65–100)	79.4 ± 7.2 80 (65–97)	82.9 ± 5.4 84 (73–92)	84.5 ± 7.3 85 (65–96)	< 0.001
**Education (years)**	5.0 ± 4.5 5 (0–18)	4.3 ± 4.2 5 (0–17)	4.4 ± 3.9 5 (0–16)	4.3 ± 4.3 5 (0–16)	4.1 ± 4.1 5 (0–16)	4.9 ± 4.25 5 (0–16)	4.3 ± 4.72 4 (0–18)	0.172
**Hypertension**	67.4%	75.6%	84.0%	66.9%	77.6%	64.6%	80.0%	< 0.001
**Diabetes Mellitus**	29.1%	43.8%	42.3%	32.5%	40.0%	36.5%	44.0%	< 0.001
**CAD**	8.6%	12.4%	6.7%	15.3%	9.5%	16.5%	12.0%	0.114
**COPD**	5.1%	4.4%	6.7%	4.0%	9.0%	6.3%	8.0%	0.314
**CVD**	6.0%	10.6%	21.3%	17.7%	11.9%	19.0%	20.0%	< 0.01
**PD**	5.1%	6.6%	5.3%	16.9%	5.0%	21.5%	32.0%	< 0.001
**Osteoarthrits**	19.5%	23.3%	26.7%	13.7%	30.2%	22.8%	20.0%	< 0.001
**Dementia**	31.8%	35.6%	12.0%	82.9%	28.5%	67.1%	64.0%	< 0.001
**Nocturia**	79.3%	89.0%	97.3%	65.3%	93.5%	82.9%	87.0%	< 0.001
**Number of drugs**	5.8 ± 3.4 6 (0–16)	6.8 ± 3.5 6 (0–21)	6.4 ± 3.2 6 (0–18)	7.42 ± 3.4 7 (0–17)	7.1 ± 3.8 7.0 (0–25)	9.8 ± 3.8 9.0 (3–16)	7.6 ± 3.8 7.0 (0–18)	< 0.001
**BADL**	87.2 ± 20.1 95 (0–100)	76.5 ± 22.3 85 (0–100)	80.9 ± 17.4 85(17–100)	34.7 ± 28.0 35 (0–95)	79.1 ± 16.0 83 (5–100)	45.5 ± 23.5 48 (0–88)	49.4 ± 23.1 53 (15–95)	< 0.001
**IADL**	13.9 ± 7.6 15 (0–23)	12.2 ± 7.61 13 (0–24)	15.2 ± 6.8 16 (0–23)	12.39 ± 4.11 11 (0–23)	13.2 ± 7.0 14 (0–51)	3.9 ± 4.8 2 (0–21)	5.1 ± 5.5 3 (0–19)	< 0.001
**Tinetti Performance**	22.9 ± 7.5 27 (0–28)	20.5 ± 8.42 24 (0–28)	21.0 ± 8.3 25 (0–28)	9.01 ± 9.2 8 (0–28)	21.4 ± 7.2 24 (0–28)	10.3 ± 9.4 9 (0–28)	10.7 ± 9.1 11 (0–28)	< 0.001
**SARC-F**	3.4 ± 3.1 2 (0–10)	4.7 ± 3.1 5 (0–10)	4.2 ± 2.9 4 (0–10)	7.4 ± 2.6 8 (0–10)	4.8 ± 2.8 5 (0–10)	7.6 ± 2.2 8 (0–10)	7.6 ± 2.2 9 (3–10)	< 0.001
**Handgrip Strength (kg)**	17.0 ± 6.5 17 (0–42)	16.1 ± 5.9 16 (0–42)	19.0 ± 16.7/18 (0–154)	8.6 ± 6.3 9 (0–24)	16.0 ± 5.6 16 (0–34)	11.1 ± 6.9 10 (0–26)	13.1 ± 6.4 12 (1–29)	< 0.001
**Fall History** **(1 year)**	36.4%	50.4%	49.3%	47.9%	49.8%	60.8%	72.0%	< 0.001
**MMSE**	21.9 ± 6.2 23 (0–30)	21.6 ± 6.41 23 (0–30)	24.6 ± 3.8 25 (13–30)	12.0 ± 8.6 12 (0–29)	22.7 ± 5.3 24 (2–30)	19.3 ± 5.4 19 (9–28)	16.2 ± 7.93 17 (0–28)	< 0.001
**GDS**	4.7 ± 3.9 4 (0–15)	5.2 ± 3.9 4 (0–15)	6.2 ± 4.2 6 (0–14)	5.7 ± 4.5 6 (0–15)	6.8 ± 4.0 6 (0–15)	8.2 ± 3.5 9 (0–15)	6.9 ± 4.3 6 (0–15)	< 0.001
**ISI**	11.1 ± 9.4 8 (0–28)	12.2 ± 9.4 10 (0–28)	11.3 ± 9.8 8 (0–28)	10.5 ± 10.2 6.00 (0–28)	13.0 ± 9.24 12 (0–28)	14.2 ± 11.2 16 (0–28)	12.05 ± 9.67 10 (0–28)	0.044
**ESS**	4.0 ± 4.7 2 (0–24)	5.3 ± 5.2 4 (0–24)	3.9 ± 4.5 3 (0–24)	8.9 ± 6.7 8 (0–24)	4.8 ± 4.9 3 (0–24)	8.0 ± 5.4 8 (0–21)	7.7 ± 5.7 7 (0–24)	< 0.001
**MNA**	21.6 ± 5.1 22.5 (4–30)	21.6 ± 4.7 22.5 (4–30)	21.5 ± 4.7 22 (3–29)	16.8 ± 5.5 17.50 (2–26)	21.7 ± 4.3 22.5 (2–29)	18.1 ± 5.0 19 (5–27.5.5)	18.4 ± 4.2 19 (9–25.5.5)	< 0.001

BADL: Basic Activities of Daily Living, CAD: Coronary Artery Disease, COPD: Chronic Obstructive Pulmonary Disease, CVD: Cerebrovascular disease, ESS: Epworth Sleepiness Scale, GDS: Geriatric Depression Scale, IADL: Instrumental Activities of Daily Living, ISI: Insomnia Severity Index, MMSE: Mini-Mental State Examination, MNA: Mini Nutritional Assessment, PD: Parkinson’s Disease, UI: Urinary incontinence, SARC-F: Strength, Assistance, Rise, Climb, and Falls score

Table 2 shows the multivariable logistic regression analysis. In multivariable logistic regression analysis, each 1-point increase in the BADL score showed a statistically significant protective effect across all types of incontinence. The odds ratios (OR) were calculated as follows: for FUIFI, OR = 0.789 (CI: 0.71–0.86; *p* < 0.001); for UUI, OR = 0.908 (CI: 0.88–0.98; *p* < 0.001); for SUI, OR = 0.915 (CI: 0.87–0.95; *p* < 0.001); for MUI, OR = 0.913 (CI: 0.88–0.94; *p* < 0.001); and for UI + FI, OR = 0.861 (CI: 0.81–0.91; *p* < 0.001). Accordingly, each 1-point increase in the BADL score reduces the risk of UUI by 9% and the risk of FUI by 21%. An increase in the Lawton score was significantly associated with increased risk only in the MUIgroup, with OR = 1.112 (CI: 1.03–1.19; *p* = 0.006), indicating that each 1-point increase in the Lawton score raises the risk of MUI by 11%. Regarding polypharmacy, the average number of medications used was significantly higher in the FUIand UUI + FUI groups (*p* < 0.001). In the UUI + FUI group, 72% of individuals used at least five medications, compared to 36% in the control group. However, in the multivariable analysis, the total number of medications was not identified as an independent significant variable. Handgrip strength showed a significant protective effect only in the FUI group (OR = 0.664; CI: 0.48–0.90; *p* = 0.010); in this group, each 1 kg increase in hand strength reduced the likelihood of incontinence by 34%. Although similar trends were observed in other groups, statistical significance was not achieved. The presence of nocturia was especially prevalent in the UUI, SUI, and UUI + SUI groups. The frequency of nocturia was found to be 97.3% in the SUI group, 89.0% in the UUI group, 93.5% in the UUI + SUI group, 82.9% in the UUI + FUI group, 87.0% in the SUI + FUI group, 65.3% in the FUI group, and 79.3% in the control group. The differences between groups were statistically significant (*p* < 0.001). The absence of nocturia was found to be significantly protective in the UUI (OR = 0.183; CI: 0.083–0.40; *p* < 0.001), SUI (OR = 0.092; CI: 0.009–0.93; *p* = 0.044), and UUI + SUI (OR = 0.228; CI: 0.07–0.67; *p* = 0.008) groups. The absence of hypertension was identified as a significant risk factor only in the FUI subtype (OR = 31.162; CI: 1.14–848.7; *p* = 0.041); no significant associations were observed in the other types of incontinence.The absence of stroke (CVA) history was significantly protective only in the SUI group (OR = 0.157; CI: 0.043–0.57; *p* = 0.005); no similar effects were noted in other subtypes.The absence of dementia was identified as a significant protective factor for UUI + FUI (OR = 0.087; CI: 0.016–0.47; *p* = 0.005). A similar trend was observed in the FUI group, though not statistically significant (OR = 0.085; CI: 0.005–1.48; *p* = 0.091). Age was found to be a significant protective factor only for the FUI group, with each one-year increase reducing the likelihood of developing FUI by 26% (OR = 0.744; CI: 0.58–0.93; *p* = 0.013). An increase in BMI was identified as a significant risk factor only in the UUI + SUI group (OR = 1.090; CI: 1.01–1.18; *p* = 0.021); no significant associations were observed in other subgroups. HbA1c level was significantly associated with increased risk only in the UUI + SUI group (OR = 1.421; CI: 1.02–1.96; *p* = 0.034). Additionally, an increase in glomerular filtration rate (GFR) was identified as a significant protective factor in the UUI + FUI group (OR = 0.954; CI: 0.91–0.99; *p* = 0.028).

**Table 2:** Multinomial logistic regression

Variables	Urge UI OR (95%CI)/*p*	Stress UI OR (95%CI)/*p*	Functional UI OR (95%CI)/*p*	Urge&Stress UI OR (95%CI)/*p*	Urge&Functional UI OR (95%CI)/*p*
**Age**,** years**	0.993 (0.95–1.03)/*p* = 0.759	0.984 (0.90–1.06)/*p* = 0.683	0.744 (0.58–0.93)/*p* = 0.013	1.023 (0.96–1.08)/*p* = 0.462	0.986 (0.87–1.10)/*p* = 0.808
**Dementia**	0.797 (0.40–1.57)/*p* = 0.513	2.41 (0.54–10.59)/*p* = 0.244	0.085 (0.005–1.48)/*p* = 0.091	0.513 (0.21–1.20)/*p* = 0.124	0.087 (0.016–0.47)/*p* = 0.005
**Hypertension**	1.438 (0.80–2.56)/*p* = 0.220	0.315 (0.80–1.23)/*p* = 0.098	31.162 (1.14–848.74.14.74)/*p* = 0.041	1.017 (0.46–2.23)/*p* = 0.967	3.404 (0.74–15.47)/*p* = 0.113
**Diabetes Mellitus**	0.684 (0.37–1.25)/*p* = 0.219	0.781 (0.26–2.32)/*p* = 0.657	2.250 (0.14–36.11)/*p* = 0.567	1.378 (0.59–3.16)/*p* = 0.450	5.487 (0.70–42.97.70.97)/*p* = 0.105
**Cerebrovascular disease**	0.437 (0.15–1.20)/*p* = 0.108	0.157 (0.43–0.57)/*p* = 0.005	0.354 (0.01–12.69)/*p* = 0.570	0.941 (0.23–3.85)/*p* = 0.932	0.585 (0.46–7.52)/*p* = 0.681
**Osteoarthrits**	1.02 (0.59–1.77)/*p* = 0.923	0.758 (0.27–2.06)/*p* = 0.588	6.15 (0.53–72.43)/*p* = 0.146	1.394 (0.67–2.88)/*p* = 0.371	1.552 (0.33–7.19)/*p* = 0.574
**Nocturia**	0.183 (0.83 − 0.40)/*p* < 0.001	0.092 (0.009–0.93)/*p* = 0.044	0.088 (0.005–1.61)/*p* = 0.102	0.228 (0.07–0.67)/*p* = 0.008	0.317 (0.048–2.07)/*p* = 0.231
**Number of drugs**	0.908 (0.88–0.93)/*p* = 0.175	0.902 (0.77–1.05)/*p* = 0.189	1.25 (0.85–1.83)/*p* = 0.255	0.985 (0.89–1.08)/*p* = 0.762	1.00 (0.80–1.24)/*p* = 0.996
**BADL**	0.908 (0.80.98)/*p* < 0.001	0.915 (0.87–0.95)/*p* < 0.001	0.789 (0.71–0.86)/*p* < 0.001	0.913 (0.88–0.94)/*p* < 0.001	0.861 (0.81–0.91)/*p* < 0.001
**IADL**	1.044 (0.98–1.109)/*p* = 0.155	1.09 (0.98–1.20)/*p* = 0.091	1.09 (0.92–1.30)/*p* = 0.293	1.112 (1.03–1.19)/*p* = 0.006	1.00 (0.87–1.16)/*p* = 0.904
**Tinetti Performance**	1.00 (0.94–1.05)/*p* = 0.996	0.933 (0.85–1.02)/*p* = 0.131	1.20 (0.98–1.48)/*p* = 0.071	1.01 (0.94–1.08)/*p* = 0.734	1.055 (0.92–1.19)/*p* = 0.409
**Handgrip Strength (kg)**	0.992 (0.94–1.04)/*p* = 0.777	1.051 (0.99–1.11)/*p* = 0.105	0.664 (0.48–0.90)/*p* = 0.010	1.00 (0.94–1.07)/*p* = 0.892	1.047 (0.96–1.14)/*p* = 0.300
**Fall history in 1 year**	0.726 (0.43–1.21)/*p* = 0.222	0.528 (0.20–1.38)/*p* = 0.194	1.128 (0.15–8.34)/*p* = 0.906	1.117 (0.56–2.20)/*p* = 0.750	0.405 (0.10–1.64)/*p* = 0.206
**TUG (second)**	0.993 (0.97–1.01)/*p* = 0.484	0.973 (0.92–1.02)/*p* = 0.282	1.064 (1.00–1.13.00.13)/*p* = 0.045	0.997 (0.97–1.02)/*p* = 0.803	1.025 (0.99–1.06)/*p* = 0.158
**GDS**	1.00 (0.935–1.08)/*p* = 0.814	1.09 (0.96–1.25)/*p* = 0.166	0.684 (0.45–1.03)/*p* = 0.075	1.138 (1.03–1.25)/*p* = 0.010	0.999 (0.83–1.20)/*p* = 0.994
**ISI**	0.988 (0.95—1.01)/*p* = 0.405	0.959 (0.907–1.01)/*p* = 0.139	1.06 (0.94–1.20)/*p* = 0.289	0.979 (0.94–1.01)/*p* = 0.280	0.961 (0.88–1.04)/*p* = 0.322
**BMI**	1.055 (0.99–1.12)/*p* = 0.076	0.980 (0.86–1.10)/*p* = 0.746	1.057 (0.78–1.42)/*p* = 0.714	1.09 (1.01–1.18)/*p* = 0.021	1.037 (0.90–1.18)/*p* = 0.597
**MNA**	1.012 (0.94–1.08)/*p* = 0.757	0.983 (0.87–1.10)/*p* = 0.772	1.480 (1.01–2.15)/*p* = 0.042	0.925 (0.90–1.09)/*p* = 0.925	1.014 (0.82–1.24)/*p* = 0.895
**GFR (mL/dak/1.73 m²)**	1.00 (0.98-0.102.98.102)/*p* = 0.549	1.00 (0.97–1.02)/*p* = 0.986	1.01 (0.94–1.08)/*p* = 0.782	1.011 (0.99–1.03)/*p* = 0.279	0.954 (0.91–0.99)/*p* = 0.028
**HbA1c (%)**	1.234 (0.93–1.62)/*p* = 0.131	1.21 (0.76–1.91)/*p* = 0.409	1.229 (0.42–3.53)/*p* = 0.702	1.421 (1.02–1.96)/*p* = 0.034	1.821 (0.97–3.41)/*p* = 0.062
**Folate (ng/mL)**	0.988 (0.92–1.05)/*p* = 0.0701	0.954 (0.84–1.08)/*p* = 0.461	1.054 (0.85–1.30)/*p* = 0.625	1.01 (0.93–1.10)/*p* = 0.693	1.118 (0.97–1.27)/*p* = 0.101

BADL: Basic Activities of Daily Living BMI: Body Mass Index, GDS: Geriatric Depression Scale, GFR: glomerular filtration rate, IADL: Instrumental Activities of Daily Living, ISI: Insomnia Severity Index, MNA: Mini Nutritional Assessment, TUG: Timed Up and Go test, UI: Urinary incontinence

(Reference category for the dependent variable: Control group (continence – no urinary incontinence). Binary categorical predictors were coded as “absent = 0/present = 1.” Odds ratios (ORs) are reported for the “present” category compared to the reference (“absent”) category. For continuous variables, ORs are reported per one-unit increase.)

Post-Hoc analysis was shown in Supplementary Table 1.

## Discussion

It is well established that UI plays an active role in the development of dependency. The association between UI and functional capacity loss can be explained through multiple pathophysiological and behavioral mechanisms. These include: the increased risk of falls and fractures caused by UI leading to reduced physical activity and functional decline; the reciprocal effect where diminished physical capacity contributes to the onset of UI; the presence of shared risk factors for both conditions (such as white matter changes, stroke, and other neurological disorders); and the conceptualization of UI within the multifactorial etiology of geriatric syndromes. Moreover, UI is also regarded as a marker of frailty [23]. In this context, the findings of our study demonstrate that each one-point increase in the BADL score exerts a statistically significant protective effect against all types of incontinence, underscoring the critical role of functional independence in determining the risk of UI. Although immobility has been widely recognized in the literature as a risk factor for UUI, MUI, and other subtypes of UI [24, 25], several studies have reported comparable BADL scores among individuals with and without UI complaints [26, 27]. Notably, Pizzi et al. observed a significant difference in functional status between continent and incontinent individuals when assessed using the Functional Independence Measure (FIM) [25], but this difference was not evident when using the BADL. The authors attributed this discrepancy to the lower sensitivity of the BADL in detecting more subtle gradations in functional capacity [27, 28]. Consistent with this perspective, our findings further suggest that dependency—rather than chronological age—is significantly associated with all forms of UI. This highlights that functional capacity may play a more pivotal role than age alone in the pathogenesis of UI. Specifically, our data reveal that for every one-point increase in the BADL score, the risk of UUI decreases by 9%, while the risk of FUI decreases by 21%. These results imply that enhancing functional independence may not only contribute to a better quality of life but may also serve as a key preventive strategy in the management of UI.

Although nocturia is a highly prevalent symptom in older adults, comprehensive and reliable data on its association with UI and its subtypes remain limited in the existing literature. Most previous studies tend to focus on nocturia in isolation, often without simultaneous assessment of UI, making it difficult to disentangle the specific contributions of each condition. In this regard, our study provides valuable insights by examining the co-occurrence of nocturia across different UI subtypes. We found that the frequency of nocturia was remarkably high across all groups, with the highest prevalence observed in the SUI group (97.3%), followed by the UUI + SUI group (93.5%), UUI group (89.0%), SUI + FUI group (87.0%), UUI + FUI group (82.9%), and FUI group (65.3%). In comparison, the control group demonstrated a nocturia prevalence of 79.3%. The differences between groups were statistically significant. These findings suggest that nocturia is not only common among older adults but is also more prevalent in those with various forms of UI, particularly SUI and its combinations. Our results underscore the importance of evaluating nocturia in conjunction with incontinence subtypes in geriatric assessments, as overlooking their interaction may lead to incomplete clinical interpretation and suboptimal management strategies.

UI is a highly distressing and socially stigmatizing condition that significantly impairs quality of life. Beyond its physical impact, UI is often associated with psychological consequences such as depression, particularly in older adults and those with multiple comorbidities. In line with previous studies reporting the highest prevalence of depression in women with MUI, our study also demonstrated a stronger association between MUI and depressive symptoms [29, 30]. While patients with isolated incontinence may manage their symptoms with compensatory strategies that support daily functioning, the more complex nature of MUI may pose greater challenges and contribute to an increased risk of depression.

UI is a well-known clinical condition following stroke, with an incidence ranging from 37% to 79%2 [27, 31, 32]. Detrusor overactivity (DO) appears to be the most common mechanism underlying UI after stroke, although cases with underactive detrusor and normal urodynamic patterns have also been reported [33]. In contrast to previous studies, our findings reveal a significantly higher incidence of SUI post-stroke (*p* = 0.005). This difference may be attributed to the unique characteristics of our sample or methodological variations. Furthermore, it remains a topic of controversy whether cerebral laterality plays a role in bladder and sphincter function [34]. An additional factor that may have influenced our findings is the lack of distinction between patients who had pre-existing UI and those who developed newly diagnosed UI after stroke. Not separately evaluating these two groups could have contributed to the differences in outcomes observed in our study. Our findings demonstrated that, among the comorbid conditions assessed, high BMI and poor glycemic control—as indicated by elevated HbA1c levels—were significantly associated with a greater prevalence of MUI. While Thai et al. did not identify obesity as a significant risk factor for UI [35], our results align with other studies reporting a strong positive association between higher BMI and the prevalence of MUI [36, 37]. Although previous research has yielded conflicting results regarding the relationship between glycemic control and UI, our study supports a significant link between elevated HbA1c levels and MUI. This finding is consistent with previous researchs, suggesting that poor glycemic control may play a role in the development or progression of MUI [38, 39].

In studies that included both sexes, the prevalence of FUI ranged from 21.4% to 56.1%, whereas in populations consisting exclusively of women, it ranged from 2% to 2.88% [6, 40–42]. This discrepancy may be attributed to differences in gender composition and mean age across the study populations. In our study, the prevalence of FUI was 7.6%, which is higher than the rates reported in previous studies conducted exclusively among female populations (2% in a study with 42 participants and 2.88% in another with 300 participants) [6, 42]. The relatively small sample sizes in those studies may partly explain the discrepancy. We observed that FUI was associated with increased dependency, malnutrition, muscle strength loss, sarcopenia, and cognitive impairment. These findings are, to some extent, expected. Additionally, FUI was more prevalent among patients with a history of falls, balance impairments, and gait disturbances—also anticipated associations given the nature of the condition. Interventions targeting nutritional support, muscle strengthening, and treatment of sarcopenia may contribute to the improvement of FUI and, more broadly, UI. Previous studies have shown a significant relationship between cognitive and physical function decline and UI in older women [43–45]. These studies emphasize the strong association between UI and impairments in cognitive tasks related to prefrontal cortex function; however, they do not include a specific evaluation of FUI. Sarcopenia was another factor found to be associated with FUI in our study. Prior investigations have demonstrated a strong link between UI and musculoskeletal dysfunction affecting mobility and physical performance in older adults [46, 47]. Reduced pelvic floor muscle strength and diminished functional independence appear to contribute to the development of FUI. Therefore, considering all these studies, it is not surprising that FUI is associated with numerous geriatric syndromes, and adopting a comprehensive geriatric approach is essential in the management of patients with FUI.

One limitation of our study is the lack of evaluation of other known risk factors for UI, such as multiparity, mode of delivery, pelvic surgery, radiation, and systemic hormonal replacement therapy. Another limitation is that this study is a gender- and age-specific retrospective analysis and thus limits generalisability to males and other age groups. Last, we did not evaluate overflow incontinence and there was a small number of co-existence of SUI and FUI; thus, we could not analyse these subtypes. Our study has some strengths. A large sample size, conducting a CGA, and being one of the few studies that raise awareness about FUI.

The current management of UI in older women often fails to recognize the presence of FUI and its associated factors, potentially leading to the neglect of non-urological contributors to UI as part of a geriatric syndrome in this population. However, FUI is associated with several geriatric conditions, including increased functional dependency, nutritional deterioration, reduced muscle strength, and impaired balance and gait functions. Therefore, when approaching an older female patient with incontinence, it is essential to be aware of FUI, rather than focusing solely on UUI or SUI. Since successful aging strategies can reduce geriatric syndromes, they may also be effective in controlling FUI.

## Supplementary Information

Below is the link to the electronic supplementary material.

Supplementary Material 1

## Data Availability

Data will be avaliable if requested from corresponding author.
