# Supplementary material for: The prevalence of functional urinary incontinence and its association with comprehensive geriatric assessment parameters in older women
Source: Aging Clin Exp Res. 2025 Nov 14;37(1):325. doi: 10.1007/s40520-025-03228-9 (PMC12618406; doi:10.1007/s40520-025-03228-9)
Supplement: Supplementary file 1 — Supplementary Material 1 [file 40520_2025_3228_MOESM1_ESM.docx]

**Supplemantary Table 1. Post-hoc Analysis**

|  | **Age** | **Number of medication** | **SARC-F** | **BADL** | **IADL** | **MNA** | **Tinetti Total** | **GDS** | **MMSE** | **ESS** | **ISI** | **Education** |
| --- | --- | --- | --- | --- | --- | --- | --- | --- | --- | --- | --- | --- |
| **Control vs UI** | 0.094 | 0.000 | <0.001 | <0.001 | 0.000 | 0.787 | 0.000 | 0.065 | 0.457 | <0.001 | 0.043 | 0.173 |
| **Control vs SI** | 0.260 | 0.237 | 0.043 | <0.001 | 0.228 | 0.656 | 0.110 | 0.005 | 0.001 | 0.896 | 0.825 | 1000 |
| **Control vs FI** | 0.000 | 0.000 | <0.001 | <0.001 | <0.001 | <0.001 | 0.000 | 0.105 | <0.001 | <0.001 | 0.301 | 0.342 |
| **Control vs UI & SI** | 0.712 | 0.000 | <0.001 | <0.001 | 0.092 | 0.216 | 0.001 | <0.001 | 0.185 | 0.035 | 0.009 | 0.829 |
| **Control vs UI & FI** | 0.000 | 0.000 | <0.001 | <0.001 | <0.001 | <0.001 | <0.001 | <0.001 | <0.001 | <0.001 | 0.451 | 0.219 |
| **Control vs SI & FI** | 0.025 | 0.000 | <0.001 | <0.001 | <0.001 | 0.001 | <0.001 | <0.001 | 0.013 | <0.001 | 0.215 | 0.109 |
| **UI vs SI** | 0.054 | 0.276 | 0.157 | 0.258 | 0.003 | 0.756 | 0.343 | 0.060 | <0.001 | 0.061 | 0.698 | 0.847 |
| **UI vs FI** | 0.000 | 0.070 | <0.001 | <0.001 | 0.000 | 0.000 | 0.000 | 0.448 | <0.001 | <0.001 | 0.028 | 0.219 |
| **UI vs UI & SI** | 0.117 | 0.448 | 0.677 | 0.779 | 0.300 | 0.987 | 0.594 | <0.001 | 0.066 | 0.333 | 0.271 | 0.967 |
| **UI vs UI& FI** | 0.000 | 0.105 | 0.829 | <0.001 | <0.001 | <0.001 | <0.001 | 0.002 | <0.001 | <0.001 | 0.799 | 0.915 |
| **UI vs SI & FI** | 0.081 | 0.000 | 0.004 | <0.001 | <0.001 | 0.001 | <0.001 | <0.001 | <0.001 | 0.009 | 0.525 | 0.511 |
| **SI vs FI** | 0.000 | 0.031 | <0.001 | <0.001 | <0.001 | <0.001 | <0.001 | 0.436 | <0.001 | <0.001 | 0.375 | 0.677 |
| **SI vs UI & SI** | 0.426 | 0.144 | 0.122 | 0.228 | 0.036 | 0.784 | 0.593 | 0.223 | 0.023 | 0.258 | 0.174 | 0.840 |
| **SI vs UI & FI** | 0.000 | 0.040 | <0.001 | <0.001 | <0.001 | <0.001 | <0.001 | 0.310 | <0.001 | <0.001 | 0.698 | 0.470 |
| **SI vs SI & FI** | 0.010 | 0.000 | <0.001 | <0.001 | <0.001 | 0.004 | <0.001 | 0.027 | <0.001 | 0.001 | 0.332 | 0.389 |
| **FI vs UI & SI** | 0.000 | 0.300 | <0.001 | <0.001 | <0.001 | <0.001 | <0.001 | 0.037 | <0.001 | <0.001 | 0.007 | 0.619 |
| **FI vs UI & FI** | 0.491 | 0.919 | 0.654 | 0.413 | 0.128 | 0.219 | 0.583 | 0.082 | 0.021 | 0.628 | 0.181 | 0.413 |
| **FI vs SI& FI** | 0.727 | 0.013 | 0.710 | 0.342 | 0.058 | 0.512 | 0.701 | <0.001 | <0.001 | 0.966 | 0.103 | 0.508 |
| **UI& SI vs**  **UI & FI** | 0.000 | 0.317 | <0.001 | <0.001 | <0.001 | <0.001 | 0.002 | 0.007 | 0.000 | <0.001 | 0.357 | 0.473 |
| **UI & SI vs**  **SI & FI** | 0.021 | 0.000 | <0.001 | <0.001 | <0.001 | 0.001 | <0.001 | 0.099 | 0.004 | <0.001 | 0.862 | 0.521 |
| **UI & FI vs SI& FI** | 0.444 | 0.021 | 0.942 | 0.696 | 0.392 | 0.896 | 0.998 | 0.140 | 0.321 | 0.778 | 0.483 | 0.542 |

**p-values represent Kruskal-Wallis tests for continuous variables.**

FI: Functional Incontinence SI: Stress Urinary Incontinence UI: Urge Urinary Incontinence

BADL: Basic Activities of Daily Living, CAD: Coronary Artery Disease, COPD: Chronic Obstructive Pulmonary Disease, CVD: Cerebrovascular disease, ESS: Epworth Sleepiness Scale, GDS: Geriatric Depression Scale, IADL: Instrumental Activities of Daily Living, ISI: Insomnia Severity Index, MMSE: Mini-Mental State Examination, MNA: Mini Nutritional Assessment, PD: Parkinson’s Disease, UI: Urinary incontinence, SARC-F: Strength, Assistance, Rise, Climb, and Falls score
